# Sweet dreams? Testing prognostics and reinterpreting sugarcane ethanol biofuel in Brazil

**DOI:** 10.1080/25729861.2024.2398950

**Published:** 2024-12-02

**Authors:** André Sica de Campos, David Tyfield, Leonardo Freire de Mello, Brian Garvey

**Affiliations:** a School of Applied Sciences, Universidade Estadual de Campinas (Unicamp), Limeira, Brazil; b Lancaster Environment Centre, Lancaster University, Lancaster, UK; c Centre for Engineering, Modelling and Applied Social Sciences, ABC Federal University, São Bernardo do Campo, Brazil; d Department of Work, Employment and Organisation, University of Strathclyde, Glasgow, UK

**Keywords:** Sugarcane ethanol, biofuels, responsible research and innovation, power/knowledge, Etanol de cana-de-açúcar, biocombustíveis, inovação e pesquisa responsável, poder/conhecimento, Etanol de caña de azúcar, biocombustibles, innovación e investigación responsable, poder/conocimiento

## Abstract

We present a framework regarding analysis of, and engagement with, socio-technical innovations and power/knowledge trajectories for sugarcane ethanol biofuel in Brazil, testing post hoc our prognostics against recent factual evidence. This assessment offers evidence regarding the value of such analysis, given the relative rarity of research that revisits anticipated changes in Science and Technology Studies. Contending that learning and improving our forms of engaged analysis demands such retrospective assessment, we report results of a 2014/2015 study on sugarcane ethanol biofuel and automobility in Brazil. In this study, we analyze the discourse of a “responsible research and innovation” (RRI) perspective and confirm particular trends in Brazil.

## RESUMO

Apresentamos um quadro analítico acerca do engajamento com inovações sociotécnicas e suas possíveis trajetórias para o etanol, testando ex-post nossos prognósticos com evidências factuais recentes. Esta avaliação oferece evidência com respeito à validade de tal análise, dada a raridade relativa de pesquisa que revisita mudanças antecipadas a partir Estudos de Ciência e Tecnologia. Partindo da premissa que aprendizagem e melhoria sobre nossa forma de análise engajada requer tal avaliação retrospectiva, apresentamos resultados originais de uma estudo de 2014/5 sobre etanol de cana-de-açúcar e automobilidade no Brasil. Neste estudo, os autores analisam o discurso de “pesquisa e inovação responsável” na perspectiva do etanol, identificam-se tendências específicas no Brasil e confirmam-se as mesmas.

## RESUMEN

Presentamos un marco de análisis sobre el compromiso con las innovaciones sociotécnicas y sus posibles trayectorias para los biocombustibles, probando ex-post nuestros pronósticos contra la evidencia fáctica reciente. Esta evaluación proporciona evidencia sobre la validez de dicho análisis, dada la relativa rareza de la investigación que revisa los cambios anticipados de los Estudios de Ciencia y Tecnología. Se argumenta que el aprendizaje y la mejora en nuestra forma de análisis comprometido requiere dicha evaluación retrospectiva, presentamos resultados no publicados de un estudio 2014/5 sobre etanol de caña de azúcar y automovilidad en Brasil. En este estudio, los autores analizan el discurso de la “innovación y la investigación responsable” desde la perspectiva del etanol, y se identifican y se confirman tendencias específicas en Brasil.

## 1. Introduction

Ethanol biofuel emerged in the 1970s because of the Brazilian leadership in sugarcane (Cortez 2016). Heralded as sustainable, it has been situated within the discourse of responsible research and innovation (RRI) (Macnaghten et al. 2014). The topic of this paper is the reinterpretation of sugarcane ethanol biofuel in Brazil focusing on automobility and building on RRI.

The problem on the sustainability of ethanol biofuels is debatable. For some, they are intensive in land and water and lead to deforestation (Sills, Ferrante, and Fearnside 2018). Furthermore, they can potentially result in a negative carbon balance (Searchinger et al. 2008), unfair labor practices (Jesus, Genevieve, and Richardson 2016), and disturbed food markets (Monteiro, Altman, and Lahiri 2012). Contenders disagree (Augusto Horta Nogueira and Silva Capaz 2013; Gauder, Graeff-Hönninger, and Claupein 2011). For them, ethanol biofuels are part of sustainable regional development strategies (Moraes, Oliveira, and Diaz-Chavez 2015), generating less (atmospheric) pollution (Bordonal et al. 2018; Coelho et al. 2006) and more jobs than other commodities (Mesquita and Furtado 2016; Moraes, Oliveira, and Diaz-Chavez 2015). This paper does not intend to solve this debate but aims to visibilize the systemic complexity of the Brazilian sugarcane ethanol biofuel. It proposes a framework to both analyze and engage with socio-technical innovations and their likely trajectories in line with propositions of RRI as governance arenas (Owen, Von Schomberg, and Macnaghten 2021).

We update results of a 2014/2015 study to analyze the discourse of RRI from the perspective of sugarcane ethanol biofuel (Owen, Von Schomberg, and Macnaghten 2021). We point out the latter’s failure, in 2015, in providing a sustainable socio-technical energy transition in Brazil from a wider automobility perspective.

After briefly introducing RRI in Section 2, Section 3 presents our methods and framework. The findings and update of our study on Brazilian biofuel as RRI are presented in Section 4. Section 5 (Conclusions) assesses the validity of our approach.

## 2. RRI and sugarcane ethanol biofuels in Brazil

We considered the significance of the RRI discourse and practice as a productive resource involved in attempted co-production of restabilized complex power/knowledge systems on a global scale (Lynch 2014). By power/knowledge systems we mean the mutual, iterative, and constitutive link between power exerted as knowledge and knowledge conceived as power (Yates 2014). Through these lenses, we propose to contribute to RRI studies with a phronetic approach (Flyvbjerg, Landman, and Schram 2013). Phronesis means situated practical wisdom and engagement with the elements that form complex systems, and the making of ethically adequate and intuitive decisions in this process (D’souza and Introna 2024). These decisions and engagement practices may help in the constructive reconfiguration of such systems, particularly in automobility (Tyfield et al. 2020).

This paper extends the RRI agenda beyond how to manage controversial innovations through stakeholder engagement (Owen, Macnaghten, and Stilgoe 2020). In relation to biofuels, RRI research has demonstrated concerns about their economic viability, hidden impacts, and limits to their use (Capurro et al. 2015; Groves, Sankar, and Thomas 2018). Previous work on participatory engagement with excluded actors demonstrated a need for cognitive openness (Valkenburg et al. 2020). In line with these and recent interpretations of RRI, we propose a phronetic approach to engage with stakeholders and extend an understanding of sugarcane ethanol biofuel (Owen, Von Schomberg, and Macnaghten 2021) in Brazil.

According to de León Escobedo (2023), this approach can contribute to an autonomous Latin American vision on sustainability issues. Additionally, there is a need to better characterize mainstream agricultural systems, so that alternatives can be devised (Levidow, Sansolo, and Schiavinatto 2021). In particular, there is a gap on how new technologies are integrated in food production (sugar in our case), particularly in terms of social, economic, and environmental issues (Vargas-Canales et al. 2022). This paper attempts to contribute to these discussions.

We illustrate the importance of examining the emerging policy discourse of RRI regarding this case of biofuels in ways that consider the open empirical question, what work that is that discourse doing in shaping socio-technical trajectories? To address this question, we propose to extend RRI analysis by drawing on an approach based on power/knowledge systems shaping uncertain technoscience (Lynch 2014).

## 3. Method and framework

Regarding data collection, we worked with participants in ongoing power/knowledge trajectories to illuminate the power relations at play. By power/knowledge trajectories we refer to the multiple, existing and potential, hierarchical and heterarchical connections and relationships between participants in complex systems (Kelly 1994), particularly, in sugarcane ethanol biofuel micro-innovation, meso-production, and macro-consumption levels. We also propose a phronetic approach, which is virtuous practice, resonating with arguments regarding the connections between RRI and a processual virtue ethics (Grinbaum and Groves 2013).

We collected data in the early to mid 2010s from the following sources in relation to: (i) the micro-innovation level, one of the authors collected systematic data for research and development groups working on ethanol and biofuels based on the official directory of the Scientific and Technological Development National Research Council (CNPq) to identify public capacity in Brazil; (ii) relating to the meso production level, one of the authors engaged extensively with workers and also with landless workers in over 80 field interviews. This provided a detailed view of industrial relations, the contradictions between environmental sustainability, the use of natural resources, and working practices. Fieldwork covered the states of São Paulo and Goiás. (iii) This data was pooled with expertise on urban automobility, looking at macro-consumption issues, by two of the authors. The co-authors integrated analysis during two working meetings in Brazil and in the UK.

Data was analyzed observing how sugarcane ethanol biofuel was framed and presented as responsible. The analysis also considered the implementation of the novel fuel and how its related “responsibility” was constructed. This made evident that “RRI” research could become a process with potential to directly intervene in its construction. Power issues directed us to international political economy. Sugarcane ethanol is situated within global production chains and related systems of capital (agribusiness in our case) accumulation and their localized contestation by workers (Bair 2005; Silver 2003). These are refered to in this paper as industrial conflicts in the context of Brazil (Macdonald and Vandenabeele 1996, 6), which tend to reproduce power asymmetries to the advantage of agribusiness (Selwyn 2012; Stewart and Garvey 2015). RRI may, at times, serve to occlude the continuing status quo of systematically and globally “organized irresponsibility” even under the banner of “development” through the propagation of inter/intra-national inequality (Beck 2009).

We attempted to explore RRI from the perspective of how “innovation presented as responsible” was actually conducted and was itself constructing the meaning of “responsible.” In global production systems, this raised a further issue: mainstream multilateral institutions view innovation and technology as ways to increase output and jobs (Godin 2009), influencing policymakers in the global South to view innovation as a way to catch-up. “Innovation” is a key locus for geopolitical competition regarding political-economic influence in the world. This works by way of the position it secures in the international division of labor of innovation, the distribution of innovation super-rents, and concomitant socio-economic development. Integration to catch-up and the challenge of system and climate crisis include localized social-system resilience and adaptation. Research and innovation will thus be “responsible” in the Global South to the extent it elicits cycles of innovation capacity and socio-economic development to tackle these systemic challenges for those who “matter,” that is, vulnerable workers (Garvey and Barreto 2016).

Brazilian sugarcane ethanol biofuel has not only been defined as part of the discourse of autonomous competitiveness by policymakers, and as technoscientific progress by scientists (Cortez 2016), it has also been presented by agribusiness as innovation that is renewable, sustainable (UNICA 2019) and (thus) responsible. Next, we synthesize these dimensions of the Brazilian sugarcane ethanol biofuel discourse.

## 4. Context and empirical findings

### 4.1. Context: innovating energy for automobility in Brazil

Automobility is a key aspect in urban forms of societal governance (Rajan 2006). A central aspect of automobility is reliance on fossil fuels (Tyfield 2014a), which of course renders it a socio-technical system that is environmentally impactful (Geels et al. 2011). Although sugarcane ethanol fuel has been heralded as a sustainable alternative, the existing fossil fuel automobility system is also amongst the most “locked-in” of high-carbon socio-technical systems (Geels et al. 2011).

Fossil fuel automobility has proved to be resilient. Even though Global North Sales have declined (Cohen 2012) this has occurred incrementally. The Global South forecast of continued significant growth of car numbers (International Energy Agency 2011) has been led by China and India (International Energy Agency 2024). In Brazil, although the production of cars has been inconsistent due to political and sanitary crises, automobility raises imaginaries of “catch-up” in production and consumption (Paterson 2007), as a way to socio-economic development. In this context, sugarcane ethanol fuel has had a continuous status of a “low-carbon” alternative to fossil fuels.

Following the power/knowledge trajectories, innovating in these energy-mobility systems is as much an issue of the systems of *consumption/demand* for automobility and the energy it consumes as of the *production/supply* and innovation of these new forms of energy source. Yet policy debates remain focused on the latter. Actual impacts – environmental, energy, resource, social – of plans for such energy innovation at the global level, and hence the innovation’s *de facto “*responsibility,” depend on how the energy produced is used, for what, and by whom.

This entails paying due attention to the parallel evolution of distinguishable sugarcane ethanol systems of micro-innovation (by scientists in Brazilian laboratories), meso-production (by agribusiness), and (urban) macro-consumption. We overview these three “moments” to furnish insights that this power/knowledge trajectories approach can yield regarding RRI.

### 4.2. “Responsible” biofuel innovation in Brazil

Sugarcane ethanol biofuel innovation sits at the heart of contemporary Brazilian power/knowledge trajectories. Policymakers created the National Alcohol Programme during the military regime to tackle the 1970s oil crisis (Bennertz and Rip 2018). The primary concern was thus Brazilian energy security (Stattman, Hospes, and Mol 2013). Framed by imaginaries of Brazilian technological upgrade and autonomy reorienting its status in the global economy around “hi-tech” agricultural products and services (Cortez 2016), sugarcane ethanol has been the focus of intense and long-standing policies (Benvenutti et al. 2023). Conceived in a top-down process, these policies were not originally or primarily substantiated in terms of the RRI agenda.

Successes in the ‘80s in constructing a sugarcane ethanol biofuel market and related innovation systems were set back in the ‘90s (Furtado, Scandiffio, and Cortez 2011). Deregulation following IMF structural adjustment and membership of the World Trade Organization (Latimer 2016) impinged on subsidies. The Planalsucar research program (which created new sugarcane seeds – “RB varieties” – that catered for a large proportion of agribusiness growth) was absorbed by federal universities with stagnation of the sugarcane ethanol market (Barbosa et al. 2012). Distilleries switched to the production of sugar for the external market (Furtado, Scandiffio, and Cortez 2011). So, it was only after the early 2000s that global leadership in the use of hydrated sugarcane ethanol as fuel emerged, prompted by flexible petrol/sugarcane ethanol engines developed by local subsidiaries of multinationals and a growing accumulation of domestic innovations by a diversified innovation system coupled with the public research system (Furtado, Scandiffio, and Cortez 2011). Agribusiness engaged in sugarcane testing/growing, more sustainable sugarcane ethanol and sugar processing, as well as machinery and technological development (Cortez 2016).

These trends created an expectation that local demand for sugarcane ethanol would increase steadily (Goldemberg, Coelho, and Guardabassi 2008) to the competitive advantage of Brazilian agribusiness innovation. The coupling of the innovation system with the production system and the expected enlarged local and international markets in the wake of the Kyoto Protocol suggested that Brazil would be able to integrate itself globally as one of the leaders in sugarcane ethanol technology. Internationally, renewed interest in sugarcane ethanol and the combination of growing demand for oil due to global economic growth with concerns about the level of greenhouse gas emissions from fossil fuels, appeared to indicate a global sugarcane ethanol market take-off (Gee and McMeekin 2011).

Sugarcane ethanol at this point began to be shaped explicitly around discourses of environmental sustainability and “responsibility.” There are two key senses to “responsible”: first, regarding the continued priority of hi-tech economic catch-up and development of the Brazilian economy (Stattman, Hospes, and Mol 2013); second, this prospect was inseparable from positive environmental credentials of biofuels against alternatives (Coelho et al. 2006; Goldemberg, Coelho, and Guardabassi 2008). These elements also fed back into the specific dominant power relations of the Brazilian innovation system. A strong internal dynamic emerged, governmental support for national elite and well-funded institutes developing hi-tech biofuel innovation in a tightly closed loop with the propagation of narratives framing biofuel’s positive prospects. This prompted investments in agro industrial capacity with state-led credit for production capacity (Furtado, Hekkert, and Negro 2020).

Had these dynamics proven productive, the discourses of sugarcane ethanol's “responsibility” in these twin senses may have proven self-sustaining. But the global sugarcane ethanol market stagnated after 2008 (Lemos et al. 2015). Locally, government policy kept petrol prices at low levels at the expense of sugarcane ethanol biofuel. The discovery of significant oil deposits off the coast of Brazil contributed to shift the geopolitical balance of power against biofuels (Furtado, Hekkert, and Negro 2020).

While EU regulations mandated increasing volumes of biofuel to be mixed in conventional automobile fuels, protests by small farmers, landless workers, and Indigenous groups sparked controversy among policy-makers in Europe, particularly regarding food vs. biofuel debates and life-cycle carbon savings (Fargione et al. 2008; Pimentel 2003), slowing the growth of this market. The sugarcane ethanol production and innovation system as a whole thus stalled, leading to a marked market concentration as companies with greater access to international finance have been able to withstand the economic instability at the expense of smaller, locally-based enterprises.

The disappointment in the growth of the Brazilian sugarcane ethanol innovation system was not indicative of a period of little qualitative change in the sector’s power relations. Over the course of the 2003–2013 period, new research institutions emerged even as traditional ones, such as the Campinas Agronomic Institute, continued to be relevant. The COPERSUCAR research centre, spun out from the cooperative and sugarcane growers, continued to cooperate with research institutions and sugarcane institutions in field tests and improvement of varieties (de Campos, Lucafó, and Corder 2015). New public-sector institutions were also created, but crucially, major multinational corporations such as BP, Endesa, Raizen, and Bunge grew significantly in the sugarcane ethanol sector, reaching over a third of the production capacity (Lemos et al. 2015). Similarly, in research Monsanto acquired two start-ups, Allelyx and CanaVialis, and started to commercialize new sugarcane varieties (Furtado, Scandiffio, and Cortez 2011); this should be a watershed in an area largely controlled by public and cooperative research. But Monsanto divested this business in 2015, arguing that the ethanol market did not return investments (NovaCana 2015).

This parallel co-evolution of discourses of “responsible” biofuel innovation and shifting power relations has so far not succeeded in sedimenting a self-sustaining common-sense of “responsibility” in the Brazilian innovation system (Mol 2010; Stattman, Hospes, and Mol 2013).

A more pronounced presence of foreign direct investment (Lemos et al. 2015) challenges the narrative of sugarcane ethanol as the vehicle for a globally competitive national innovation system. The extent to which the super-profits of innovation and influence over the Brazilian innovation system passes to corporate institutions based in the global North should be an issue of concern to local agribusiness (Mendonça, Pitta, and Xavier 2013).

Furthermore, the envisaged production of a new international commodity that is heralded as environmentally sustainable has relied to date on the intensive use of resources (UNICA 2019), including large-scale monoculture agriculture with concentrated land ownership; a model dating back to colonial Brazil (Szmrecsányi 1979). Although in terms of land extension, it is argued that sugarcane ethanol is not demanding for a country the size of Brazil (CGEE 2009), land and resource conflicts persist in the locales of its production (Kroyer 2015). Brazilian scientists have been pursuing varieties of sugarcane and processing techniques that are less intensive in natural resources (Cortez 2016). The system is locked into a large-scale and intensive production system that makes very difficult a major shift in the organization of biofuel production to more socially equitable models. The power relations of Brazilian and international agribusiness and its elite public research institutes remain tightly coupled and closed, even as both are increasingly intermediated by transnational energy and seed/agro-biotech corporations (Grandis et al. 2024). Instead of emergence of a self sustaining discourse of “RRI” in Brazil, therefore, we see feedback loops that lock-in the current system.

Post-2015 trends have not been able to revert these power-knowledge trajectories. The key issue in sugarcane ethanol has been integration in second generation technologies. Although improvements through agricultural research in sugar cane varieties have continued to advance with “c*ana energia”* (Furtado, Hekkert, and Negro 2020), a bottleneck here is the immature and internationalized nature of the technology. Much needed enzymes and processing technology have to be imported by the few pilot sites in Brazil (Lorenzi and Andrade 2019). Political crisis and ultra-liberal policies in the post-2016 affected economic growth, resulting in a stagnant car market and budgetary cuts in science and technology funding, reduced incentives for research and innovation in sugarcane ethanol.

Regarding “responsible” biofuel innovation, our analysis suggested default trajectories were unfolding toward futures in which RRI discourse was likely to support further entrenchment of existing asymmetric power relations, both intra- and inter-nationally, regarding the foregrounding of forms of “responsibility” that disproportionately benefit and support agribusiness and elite research institutions. This favored re commodification of the Brazilian economy vis-à-vis sales on the global market to richer economies. Although catching up with second generation sugarcane ethanol has proved taxing, integrating frontier agricultural and sugarcane ethanol processing knowledge, it has not diverted this trajectory. We call these “pro-elite narratives of RRI and recommodification.”

### 4.3. Labor and the internationalization of the Brazilian biofuel meso-level: production

In production, agribusiness discourse regarding limited definitions of “responsibility” failed to show the self-sustaining power/knowledge trajectory. Industrial conflicts over standards have led unions to state how the visibility of export orientated multinationals has provided leverage for them in improved site-specific health and safety (Garvey and Barreto 2014); while modernization is marketed internationally as socially and environmentally sustainable (UNICA 2019). However, looking at power dynamics entrenched in industrial conflict, concerns voiced by the workers in areas of sugarcane ethanol expansion point to increased international agribusiness control of natural resources, innovation, and market share. Three consequences ensue from deeper integration into global production chains.

First, sugar cane agribusiness competitiveness depends on continuous cost-saving, by cutting jobs (Bogdanski 2012). One crop-harvester replaces around 100 workers. It is estimated that rural manual workers will lose their jobs and rural-to-urban migration has accelerated in areas of expansion. Second, in the face of rising land rents and technical modernization from multinationals, fifty-one Brazilian agribusiness have ceased operations in the years 2007–2012 alone, cutting 45,000 direct and indirect jobs (Castilho 2012). These impacts, alongside low salaries, piece-rate and seasonal unemployment by the multinationals, and persistent breaches in employment law (Alves 2006), have meant it was increasingly difficult for the state, EU, and corporate promoters of agrofuels to sustain claims about rural development opportunities and social upgrading of workers and small land-holders (European Comission 2009; International Energy Agency 2011; International Labour Organization and United Nations Environmental Programme 2012). Workers reported precarity, exposure to agro-toxins, and concern for the air and water quality in the areas where they raise their families (Garvey and Barreto 2016).

Third, efficiency, speed, and spatial increases of resource extraction alongside renewed commodity demands have brought pioneers, Indigenous and rural populations into conflict (Antunes 2003; Gonçalves 2006) and amidst modest twenty-first century social reforms in Brazil, land inequality remains among the globe’s highest. Camps of landless workers often sit aside sites of agribusiness expansion. For example, in 2012, following pressure from Indigenous protests and the Brazilian government, Shell was forced to sign an agreement to end the sourcing of sugarcane from traditional lands of the Guarani people of Mato Grosso do Sul, drawing international attention to the ongoing land conflicts (Kroyer 2015).

Workers in sugarcane mills have not been passive agents associated with this attempted commodification of sugarcane ethanol. Resistance has been voiced through advocacy of an agrarian reform that is in direct tension with the existing model of agriculture. Particularly, industrial action ended variable salaries and performance-based pay, which exerted pressure on the productivity of manual work in specific distilleries.

A heterogeneous movement for land and agricultural reform in Brazil, the collective vision of their Federation for Rural Workers of São Paulo for food and energy production embraces novel technologies – a different vision of “innovation” and how and in what ways it could be “responsible” (Mol 2010). For them, as mechanization increases salaries but causes unemployment, organic small farming could be fostered, oriented to agroecological food production, and run by formalized workers (FERAESP 2017). This may not be RRI as defined to date in the emerging literature where sugarcane ethanol is simply an alternative, renewable, and sustainable fuel. Yet it is also a vital movement of innovation politics concerning a putatively controversial technology that is reimagining how it could be deployed “responsibly.” Phronetic engagement with these movements thus promises to illuminate definitions of RRI that do have meaningful purchase.

Even while this reimagining still acknowledges the remarkable energetic properties of the sugarcane plant, it departs radically from the hegemonic model of production that is rendering rural futures insecure. This brings to focus a point of tension – a window for phronetic enquiry (Flyvbjerg, Landman, and Schram 2013) – within the workers’ collective organization that cristallizes the broader contradictions of the sector itself. While continuing to negotiate on job quality and security at national levels, the Federation recognizes that inherent to the mode of sugarcane ethanol production is structural unemployment, the narrowing of alternative rural work opportunities, and longer term water and soil exhaustion (interviews, 2013). Hence the Federation’s energies are also spent amongst landless occupations it helps to organize on or for land thus denied to competing commercial interests (ITESP 2012).

When stepping into the encampments of semi-skilled cane harvesters and transporters, the conversation of responsible, integrated food and energy innovation shifted from one paradigm to another, in ways that may be self-enabling. The realization of such alternatives beyond isolated case studies requires technical and political engagement with workers in the co-production of knowledge of social, policy, and technical innovation. This demands a significant analytic - phronetic– shift by scientists and policymakers that are prepared to measure the viability and longer-term contribution of agribusiness across a range of biophysical, economic, and social indicators, while no longer taking for granted the deeply uneven socio-political systems within which key stakeholders are embedded. Entwined with systems of demand, it would also need political-technological change in existing locked-in models of consumption of biofuels.

In 2016 the labor-led collation government that ruled Brazil democratically since 2003 was deposed, resulting in ultra-Neoliberal policies detrimental to labor relations and the environment (Stewart et al. 2021). Hence, none of the elements described above changed structurally. Manual jobs continued to decrease (Mantovani, Shikida, and Gomes 2021). Production of sugarcane ethanol stalled nation-wide, but continued to grow in the new Cerrado biome frontier (Companhia Nacional de Abastecimento N/D). Lack of regulation has led to an increase of food prices nationally during the Pandemic. This rendered the production of sugar attractive to mills (VIDAL 2021). The new oligopolistic structure of the market with a third of the sugar cane processing under the control of international companies stabilized.

We noted that deepening the production of sugarcane ethanol reinforcing re commodification occurred while agribusinesses internationalized. This has created a stable oligopoly with the participation of new international firms, deepening integration in global production chains. Due to the stagnant performance of sugarcane ethanol biofuel production in particular, it could not contribute to deter a broader deindustrialization process. We also noted that sugarcane ethanol biofuel on its own did not prevent or alleviate rural poverty, overexploitation, and displacement (Stewart et al. 2021). Of course, one cannot blame sugarcane ethanol for the 2016/2022 explosion of poverty in Brazil. But our assertion has been in line with recent studies indicating that areas of recent expansion in sugarcane ethanol production are also more vulnerable to cyclical crisis and variable commodity prices in economic-occupational terms. Mechanized cropping eliminated unskilled jobs affecting the most vulnerable more intensely (Faria- dos-Santos et al. 2022). We label this a process of “internationalization of agribusiness and localization of land and industrial conflicts.”

### 4.4. Macro-consumption: biofuel-based automobility and urbanization in Brazil

Since 1955, the importance of automobility as a key Brazilian political project deepened. The automotive industry became increasingly important to economic development and as part of the broader project of class power shaping/disciplining of Brazilian society, again as “modern” as opposed to “backward” and “poor.” Such developments went hand-in-hand with the take-off of Brazilian mega-urbanization (Martine and McGranahan 2014), as well as the growing car industry and car consumer-citizens as a powerful lobby (Kuhnimhof et al. 2014). This included automobile workers, who ultimately challenged and overthrew the military regime. Emblematic of this continued commitment to the “car,” President Lula used the increase in car sales in his presidency as indicative of its success (Kuhnimhof et al. 2014).

Commitment to the car system continued, as sugarcane ethanol biofuel has been redefined as “responsible” consumption in the Brazilian context and as bearer of urban “green” development. Yet this model of automobility is actually problematic on its own terms and in ways that do little to address transforming the energy source from petrol to sugarcane ethanol. Sugarcane ethanol thus seems to be “responsible” in a meaningful sense within existing “RRI” literature only to the extent that it is associated with transition to a dramatically different model of urban mobility (Tyfield 2014b).

Currently, 90% of Brazilians live and work in urban areas. Car ownership has grown even faster in recent decades, at nearly 40% in metropolitan regions between 2000 and 2010 (Pereira and Schwanen 2013), to the point that it reached saturation. Traffic congestion is a crucial feature of contemporary urban Brazil. Commuter traffic is amongst the worst globally (Facchini, Bovo, and De Moraes 2014; Silva 2013). Underinvestment regarding public and mass transportation excludes road infrastructures, which are significant and prioritized investment (Lara 2012).

Mobility is increasingly a grievance in contemporary Brazil, at the same time that the car and sugarcane ethanol biofuel have been part of concerted institutional discourse of “middle class” progress that feeds this imaginary of “automobility” as development. These intensifying social tensions play out as a major aspect of the current highly contested socio-political climate. Automobility remains a site of Brazil’s intense class contestation, distance, and mutual suspicion.

Public transport was a combustible issue in the riots and marches of the 2013 “June Journeys” ignited by the rise of bus fares (Harvey et al. 2015). In response, the São Paulo municipality initiated some policies to reshape traffic with exclusive bicycle and bus lanes. There were vehement and powerful criticisms of these policies by car consumers. These groups cleave to cars, including as zones of safety, thus reproducing the parallel but separate worlds characteristic of Brazilian society. They also remain the beneficiaries of long-standing government policies of significant subsidies to automobility, while barely investing in public transport (Kuhnimhof et al. 2014).

This means that the promotion of “middle class” car as ownership (de Mello 2009) dovetails with a deepening popular commitment to the model of urban mobility that cannot function with increases in new cars. These developments are perceived as a challenge to the rich and privileged elites' “right to automobility” by the “middle class,” not to mention by poor workers. While many of these, in turn, are determined to defend at least their aspirations to, if not the actual material reality of, such a standard of living and mobility (de Mello 2009). Existing sugarcane ethanol consumption is driving a mobility system that is increasingly not even to the advantage of those that it is “supposed” to privilege. It feeds unfunctionally back into the socio-political conditions of intense stratification, mutual distrust, and contemptuous elite determination to control commitment to automobility.

The ongoing system failure or “gridlock” shows that a transition from automobility must happen in any case, as a matter of immanent need. This presents another strategic opening for the systemic reimagining and transformation of urban mobility in Brazil including “responsible” sugarcane ethanol biofuel. Recontextualized in this way, though, the hi-tech innovation of sugarcane ethanol is only “responsible” to the extent to which it is associated with, and part of and set within, a qualitatively different model of mobility. Here RRI has a significant opportunity to be strategically deployed, in projects of phronesis, to illuminate and engage with movements actively (Owen et al. 2013) exploring this sociotechnical system change and more equitable models of urban mobility.

With the 2016/2022 deregulation and neoliberalization, the “middle-class” car consumption “sweet dream” faded. Traffic conditions worsened as such dream has been replaced by the stark reality of young, informal, unskilled workers reliant on two wheel/automobility to work for apps (Ikuta and Monteiro 2021). Working long hours, these drivers have no expectation of a substantive and better long-term future. Not only food prices, but also fuel prices were deregulated, and followed international markets vicissitudes, increasing the cost of both sugarcane ethanol and petrol. Such app workers earn enough to barely cover monthly expenses and escape famine (Ikuta and Monteiro 2021).

Regarding social cohesion, we identified combustible fault-lines of mutual distrust between the elite, “middle classes,” and workers. The elite and “middle classes” attacked pro-poor social policies, which were impositions of the post-military 1988 Federal Constitution. These policies were equaled to socialism in Brazil. Increased access to public health and education, as well as to automobility did not contribute to temper national politics. In our analysis, we envisaged a process of “consumption, urbanization and automobility fracturing social cohesion.”

## 5. Conclusions

In this paper, we attempted to clarify the systemic complexity of Brazilian sugarcane ethanol biofuel. This paper reported results dating back to 2015, applying the power/knowledge systems approach we identified three broad trends which, when revisited, raised our attention as to prognostics that time unfortunately confirmed. In each of them, we identified the failure to define sugarcane ethanol as “responsible.” “Pro-elite narratives of RRI and re-commodification” have not been challenged. Research on first-generation sugarcane ethanol, efficient as it is, alongside work on second-generation sugarcane ethanol, has not raised socially/economically equitable research topics/aims. This benefits the power of elite scientists and agribusiness, which are all aligned with the existing RRI sustainable narrative. “Internationalization of agribusiness and localization of land and industrial conflicts” already under way in 2015 have been deepened. International agribusiness became integrated into Brazilian production, increasing power asymmetries in industrial relations in the wake of ultra-liberal pro-business labor legislation. The pattern of “consumption, urbanization and automobility fracturing social cohesion” has been ever more problematic. From the car as a consumption dream/symbol, now young drivers work for app platforms in the context of stagnant markets, and a frustrated “middle class” fuels political hatred. Across these three dimensions, we finish this paper calling for a need to rethink sugarcane ethanol biofuels through a phronetic approach, that is, engaging with those that are subordinated in power relations. Our approach drew from power/knowledge and phronetic approaches, which enabled a clear diagnostic and proposed incremental elements in terms of more emphatic and collaborative values that should orient public and private strategy. We believe that in itself, this approach is a practical contribution of this paper. Based on a single topic, we acknowledge the limitations of our findings, and believe that the study of other types of biofuels in different contexts might extend and hopefully validate our findings.

## References

[CIT0001] Alves, F. 2006. “Por que morrem os cortadores de cana?” *Saúde e sociedade* 15 (3): 90–98. 10.1590/S0104-12902006000300008

[CIT0002] Antunes, R. 2003. “The Metamorphoses and Centrality of Labour Today.” *Critique* 31 (1): 117–130. 10.1080/03017600309469469

[CIT0003] Augusto Horta Nogueira, L., and R. Silva Capaz. 2013. “Biofuels in Brazil: Evolution, Achievements and Perspectives on Food Security.” *Global Food Security* 2 (2): 117–125. 10.1016/j.gfs.2013.04.001

[CIT0004] Bair, J. 2005. “Global Capitalism and Commodity Chains: Looking Back, Going Forward.” *Competition & Change* 9 (2): 153–180. 10.1179/102452905X45382

[CIT0005] Barbosa, M. H. P., M. D. V. Resende, L. A. D. S. Dias, G. V. D. S. Barbosa, R. A. Oliveira, L. A. Peternelli, and E. Daros. 2012. “Genetic Improvement of Sugar Cane for Bioenergy: The Brazilian Experience in Network Research with RIDESA.” *Crop Breeding and Applied Biotechnology* 2 (spe): 87–98. 10.1590/S1984-70332012000500010

[CIT0006] Beck, U. 2009. “World at Risk.” *Polity* 9: 1908–1918.

[CIT0007] Bennertz, R., and A. Rip. 2018. “The Evolving Brazilian Automotive-Energy Infrastructure: Entanglements of National Developmentalism, Sugar and Ethanol Production, Automobility and Gasoline.” *Energy Research & Social Science* 41: 109–117. 10.1016/j.erss.2018.04.022

[CIT0008] Benvenutti, L. M. M., L. M. De Souza Campos, D. Vazquez-Brust, and C. Liston-Heyes. 2023. “The Changing Roles of Actors in ‘Fortuitous’ Sustainability Transitions: An Analysis of Brazil's Passenger Vehicles Fuel Technology from 1970 to 2020.” *Technological Forecasting and Social Change* 192: 122584. 10.1016/j.techfore.2023.122584

[CIT0009] Bogdanski, A. 2012. “Integrated Food–Energy Systems for Climate-Smart Agriculture.” *Agriculture & Food Security* 1 (1): 1–10. 10.1186/2048-7010-1-9

[CIT0010] Bordonal, R. D. O., J. L. N. Carvalho, R. Lal, E. B. De Figueiredo, B. G. De Oliveira, and N. La Scala. 2018. “Sustainability of Sugarcane Production in Brazil. A Review.” *Agronomy for Sustainable Development* 38 (2): 13. 10.1007/s13593-018-0490-x

[CIT0011] Capurro, G., H. Longstaff, P. Hanney, and D. M. Secko. 2015. “Responsible Innovation: An Approach for Extracting Public Values Concerning Advanced Biofuels.” *Journal of Responsible Innovation* 2 (3): 246–265. 10.1080/23299460.2015.1091252

[CIT0012] Castilho, A. 2012. “Com crise, país perde 30 usinas de cana-de-açúcar desde o ano passado.” *Folha de São Paulo*.

[CIT0013] CGEE. 2009. “Bioetanol combustível: Uma oportunidade para o Brasil.” *Centro de Gestão e Estudos Estratégicos, Brasília*, 536.

[CIT0014] Coelho, S. T., J. Goldemberg, O. Lucon, and P. Guardabassi. 2006. “Brazilian Sugarcane Ethanol: Lessons Learned.” *Energy for Sustainable Development* 10 (2): 26–39. 10.1016/S0973-0826(08)60529-3

[CIT0015] Cohen, M. J. 2012. “The Future of Automobile Society: A Socio-Technical Transitions Perspective.” *Technology Analysis & Strategic Management* 24 (4): 377–390. 10.1080/09537325.2012.663962

[CIT0016] Companhia Nacional De Abastecimento. N/D. Série Histórica de Produção de Açúcar. In: CONAB (ed.). DF.

[CIT0017] Cortez, L. A. B., ed. 2016. *Universidades e empresas: 40 anos de ciência e tecnologia para o etanol brasileiro*. São Paulo: Blucher.

[CIT0018] de Campos, A. L., B. Lucafó, and S. Corder. 2015. “Sistema de Inovação do Setor Sucroenergético no Brasil.” *Futuros do bioetanol: O Brasil na liderança*, edited by Sérgio Salles-Filho, 35–52. 1ed. São Paulo: Elsevier.

[CIT0019] de León Escobedo, T. G. 2023. “Scalar Dissonances, Knowledge-Making, Sense of Urgency, and Social Narratives About the Future. Contours of the Climate Change Debate in Latin America.” *Tapuya: Latin American Science, Technology and Society* 6 (1): 2278839. 10.1080/25729861.2023.2278839

[CIT0020] de Mello, L. F. 2009. “População, consumo e mudança climática.” *População e mudança climática: dimensões humanas das mudanças ambientais globais. Campinas, Brasília: Nepo/Unicamp, UNFPA*, 292.

[CIT0021] D’souza, S., and L. D. Introna. 2024. “Recovering Aristotle’s Practice-Based Ontology: Practical Wisdom as Embodied Ethical Intuition.” *Journal of Business Ethics* 189 (2): 287–300. 10.1007/s10551-023-05371-7

[CIT0022] European Comission. 2009. Directive 2009/28/EC of the European Parliament and of the Council of 23 April 2009 on the Promotion of the Use of Energy From Renewable Sources and Amending and Subsequently Repealing Directives 2001/77/EC and 2003/30/EC Renewable Energy Directive, O.J. L. Brussels: European Council.

[CIT0023] Facchini, E., C. R. M. Bovo, and A. C. De Moraes. 2014. “A mobilidade urbana na encruzilhada: debate inadiável, soluções urgentes.” *Revista dos Transportes Públicos-ANTP-Ano* 37: 30.

[CIT0024] Fargione, J., J. Hill, D. Tilman, S. Polasky, and P. Hawthorne. 2008. “Land Clearing and the Biofuel Carbon Debt.” *Science* 319 (5867): 1235–1238. 10.1126/science.1152747 18258862

[CIT0025] Faria-dos-Santos, H., M.-D.-A.-P. Sampaio, F. Mesquita, and M.-F.-V. Pereira. 2022. “Crise do setor sucroenergético no Brasil e a vulnerabilidade territorial dos municípios canavieiros.” *EURE (Santiago)* 48: 1–26.

[CIT0026] FERAESP. 2017. *Manifesto contra as reformas e por dignidade no campo*. Piratininga: Sindicato dos empregados rurais.

[CIT0027] Flyvbjerg, B., T. Landman, and S. Schram. 2013. “Tension Points in Real Social Science: A Response.” *British Journal of Sociology* 64 (4): 758–762. 10.1111/1468-4446.12047_4

[CIT0028] Furtado, A. T., M. P. Hekkert, and S. O. Negro. 2020. “Of Actors, Functions, and Fuels: Exploring a Second Generation Ethanol Transition from a Technological Innovation Systems Perspective in Brazil.” *Energy Research & Social Science* 70: 101706. 10.1016/j.erss.2020.101706

[CIT0029] Furtado, A. T., M. I. G. Scandiffio, and L. A. B. Cortez. 2011. “The Brazilian Sugarcane Innovation System.” *Energy Policy* 39 (1): 156–166. 10.1016/j.enpol.2010.09.023

[CIT0030] Garvey, B., and M. J. Barreto. 2014. “Changing Work and the Global Commodification of Ethanol.” *Ateliê Geográfico* 8: 51–73. 10.5216/ag.v8i1.29038

[CIT0031] Garvey, B., and M. J. Barreto. 2016. “At the Cutting Edge: Precarious Work in Brazil’s Sugar and Ethanol Industry.” In *Neoliberal Capitalism and Precarious Work*, edited by Rob Lambert and Andrew Herod, 165–199. Cheltenham: Edward Elgar Publishing.

[CIT0032] Gauder, M., S. Graeff-Hönninger, and W. Claupein. 2011. “The Impact of a Growing Bioethanol Industry on Food Production in Brazil.” *Applied Energy* 88 (3): 672–679. 10.1016/j.apenergy.2010.08.020

[CIT0033] Gee, S., and A. McMeekin. 2011. “Eco-Innovation Systems and Problem Sequences: The Contrasting Cases of US and Brazilian Biofuels.” *Industry and Innovation* 18 (3): 301–315. 10.1080/13662716.2011.561029

[CIT0034] Geels, F., R. Kemp, G. Dudley, and G. Lyons. 2011. *Automobility in Transition? A Socio-Technical Analysis of Sustainable Transport*. Oxford: Routledge.

[CIT0035] Godin, B. 2009. *Making Science, Technology and Innovation Policy: Conceptual Frameworks as Narratives*. Montreal/Quebec: Chair Fernand-Dumont sur la culture.

[CIT0036] Goldemberg, J., S. T. Coelho, and P. Guardabassi. 2008. “The Sustainability of Ethanol Production from Sugarcane.” *Energy Policy* 36 (6): 2086–2097. 10.1016/j.enpol.2008.02.028

[CIT0037] Gonçalves, C. W. P. 2006. *A globalização da natureza e a natureza da globalização*. Rio de Janeiro: Editora Record.

[CIT0038] Grandis, A., J. D. S. Fortirer, D. Pagliuso, and M. S. Buckeridge. 2024. “Scientific Research on Bioethanol in Brazil: History and Prospects for Sustainable Biofuel.” *Sustainability* 16: 4167. 10.3390/su16104167

[CIT0039] Grinbaum, A., and C. Groves. 2013. “What is ‘Responsible’ About Responsible Innovation? Understanding the Ethical Issues.” *Responsible Innovation: Managing the Responsible Emergence of Science and Innovation in Society*, edited by Richard Owen, John R. Bessant, and Maggy Heintz, 119–142. Chichester: John Wiley and Sons.

[CIT0040] Groves, C., M. Sankar, and P. J. Thomas. 2018. “Second-Generation Biofuels: Exploring Imaginaries via Deliberative Workshops With Farmers.” *Journal of Responsible Innovation* 5 (2): 149–169. 10.1080/23299460.2017.1422926

[CIT0041] Harvey, D., E. Maricato, M. Davis, R. Braga, S. Žižek, M. L. Iasi, F. Brito, C. Vainer, V. A. De Lima, and J. L. S. Maior. 2015. *Cidades rebeldes: Passe livre e as manifestações que tomaram as ruas do Brasil*. São Paulo: Boitempo Editorial.

[CIT0042] Ikuta, C. Y. S., and G. P. P. Monteiro. 2021. “Perfil dos motoboys e entregadores de mercadorias.” *Revista Ciências do Trabalho* 20: 1–11.

[CIT0043] International Energy Agency. 2011. “Future Biomass-based Transport Fuels: Summary and Conclusions from the IEA Bioenergy ExCo67Workshop.” *IEA Bioenergy: Cape Town, South Africa*.

[CIT0044] International Energy Agency. 2024. “Global EV Outlook 2024.” Oxford: processed by Our World in Data.

[CIT0045] International Labour Organisation And United Nations Environmental Programme. 2012. “Working Towards Sustainable Development: Opportunities for Decent Work and Social Inclusion in a Green Economy.” In ILO (ed.). Geneva.

[CIT0046] ITESP. 2012. *Fundação Itesp implanta Assentamento Mario Covas em São Simão* [Online]. Governo do Estado de São Paulo. Retrieved August 27, 2024, from https://www.saopaulo.sp.gov.br/sala-de-imprensa/release/fundacao-itespimplanta-assentamento-mario-covas-em-sao-simao/.

[CIT0047] Jesus, D. F., O. Genevieve, and B. Richardson. 2016. “Violations of Labour and Environmental Law by Sugarcane Mills in São Paulo State, Brazil.” *Ethical Sugar*.

[CIT0048] Kelly, M. 1994. “Foucault, Habermas, and the Self-Referentiality of Critique.” In *Critique and Power: Recasting the Focault/Habermas Debate*, edited by M. Kelly, 365–400. Cambridge, MA: The MIT Press.

[CIT0049] Kroyer, K. 2015. “Resource Conflicts Between Landholders and Indigenous People in Mato Grosso do Sul, Brazil: Policies, Sources and Consequences in a Historical Perspective.” *Revista Ñanduty* 3: 131–144.

[CIT0050] Kuhnimhof, T., C. Rohr, L. Ecola, and J. Zmud. 2014. “Automobility in Brazil, Russia, India, and China: Quo Vadis?” *Transportation Research Record* 2451 (1): 10–19. 10.3141/2451-02

[CIT0051] Lara, F. L. 2012. “O paradigma do asfalto.” *vitruvius*.

[CIT0052] Latimer, A. 2016. “The Free Trade Area of the Americas in the Long Crisis of Brazilian Labour.” In *Free Trade and Transnational Labour*, edited by Andreas Bieler, Bruno Ciccaglione, John Hilary and Ingemar Lindberg, 83–94. Abingdon: Routledge.

[CIT0053] Lemos, P., F. Mesquita, M. Dal Poz, L. Souza, and S. Salles-Filho. 2015. “Panorama e desempenho recente do setor sucroenergético: Condições para um novo ciclo.” *Futuros do bioetanol: o Brasil na liderança*, edited by Sérgio Salles-Filho (Org.), 9–33. 1ed. São Paulo: Elsevier.

[CIT0054] Levidow, L., D. Sansolo, and M. Schiavinatto. 2021. “Agroecological Innovation Constructing Socionatural Order for Social Transformation: Two Case Studies in Brazil.” *Tapuya: Latin American Science, Technology and Society* 4 (1): 1843318. 10.1080/25729861.2020.1843318

[CIT0055] Lorenzi, B. R., and T. H. N. D. Andrade. 2019. “O etanol de segunda geração no Brasil: Políticas e redes sociotécnicas.” *Revista Brasileira de Ciências Sociais* 34: e3410014. 10.1590/3410014/2019

[CIT0056] Lynch, R. A. 2014. “Foucault's Theory of Power.” In *Michel Foucault*, edited by Diana Taylor, 13–26. London: Routledge.

[CIT0057] Macdonald, D., and C. Vandenabeele. 1996. *Glossary of Industrial Relations and Related Terms*. Bangkok: International Labour Organization.

[CIT0058] Macnaghten, P., R. Owen, J. Stilgoe, B. Wynne, A. Azevedo, A. De Campos, J. Chilvers, et al. 2014. “Responsible Innovation Across Borders: Tensions, Paradoxes and Possibilities.” *Journal of Responsible Innovation* 1 (2): 191–199. 10.1080/23299460.2014.922249

[CIT0059] Mantovani, G. G., P. F. A. Shikida, and M. R. Gomes. 2021. “Diferenças salariais eo impacto da segmentação regional: Um estudo para os trabalhadores na cultura de cana-de-açúcar no período de 2012 e 2019.” *Revista de Economia e Sociologia Rural* 60: e241167. 10.1590/1806-9479.2021.241167

[CIT0060] Martine, G., and G. McGranahan. 2014. “Brazil’s Negligent Urban Transition and Its Legacy of Divided Cities.” In *Urban Growth in Emerging Economies*, edited by Gordon McGranhan and George Marting, 15–54. London: Routledge.

[CIT0061] Mendonça, M. L., F. T. Pitta, and C. V. Xavier. 2013. “The Sugarcane Industry and the Global Economic Crisis.” Transnational Institute (TNI), Network for Social Justice and Human Rights.

[CIT0062] Mesquita, F. C., and A. T. Furtado. 2016. “Expansão da agroindústria canavieira e qualificação da mão-de-obra em Goiás (2006-2013).” *Sociedade & Natureza* 28 (1): 67–81. 10.1590/1982-451320160105

[CIT0063] Mol, A. P. 2010. “Environmental Authorities and Biofuel Controversies.” *Environmental Politics* 19 (1): 61–79. 10.1080/09644010903396085

[CIT0064] Monteiro, N., I. Altman, and S. Lahiri. 2012. “The Impact of Ethanol Production on Food Prices: The Role of Interplay Between the U.S. and Brazil.” *Energy Policy* 41: 193–199. 10.1016/j.enpol.2011.10.035

[CIT0065] Moraes, M. A. F. D. D., F. C. R. D. Oliveira, and R. A. Diaz-Chavez. 2015. “Socio-Economic Impacts of Brazilian Sugarcane Industry.” *Environmental Development* 16: 31–43. 10.1016/j.envdev.2015.06.010

[CIT0066] NOVACANA. 2015. *Monsanto encerra negócios no mercado de cana-de-açúcar no Brasil* [Online]. Curitiba. Retrieved August 27, 2024, from https://www.novacana.com/noticias/monsanto-encerra-negocios-cana-de-acucar brasil-071015.

[CIT0067] Owen, R., P. Macnaghten, and J. Stilgoe. 2020. “Responsible Research and Innovation: From Science in Society to Science for Society, With Society.” *Science and Public Policy* 39 (6): 751–760. 10.1093/scipol/scs093

[CIT0501] Owen, R., J. Stilgoe, P. Macnaghten, M. Gorman, E. Fisher, and D. Guston. 2013. “A Framework for Responsible Innovation.” *Responsible Innovation: Managing the Responsible Emergency of Science and Innovation in Society*, edited by Richard Owen, John R. Bessant, and Maggy Heintz, 27–50. Chichester: John Wiley and Sons.

[CIT0068] Owen, R., R. Von Schomberg, and P. Macnaghten. 2021. “An Unfinished Journey? Reflections on a Decade of Responsible Research and Innovation.” *Journal of Responsible Innovation* 8 (2): 217–233. 10.1080/23299460.2021.1948789

[CIT0069] Paterson, M. 2007. *Automobile Politics*. Cambridge: Cambridge Books.

[CIT0070] Pereira, R. H. M., and T. Schwanen. 2013. “Tempo de deslocamento casa-trabalho no Brasil (1992–2009): Diferenças entre regiões metropolitanas, níveis de renda e sexo.” *Texto para discussão*. Brasília/DF.

[CIT0071] Pimentel, D. 2003. “Ethanol Fuels: Energy Balance, Economics, and Environmental Impacts are Negative.” *Natural Resources Research* 12 (2): 127–134. 10.1023/A:1024214812527

[CIT0072] Rajan, S. C. 2006. “Automobility and the Liberal Disposition.” *The Sociological Review* 54 (1_suppl): 113–129. 10.1111/j.1467-954X.2006.00640.x

[CIT0073] Searchinger, T., R. Heimlich, R. A. Houghton, F. Dong, A. Elobeid, J. Fabiosa, S. Tokgoz, D. Hayes, and T. H. Yu. 2008. “Use of U.S. Croplands for Biofuels Increases Greenhouse Gases Through Emissions from Land Use Change.” *Science* 319 (5867): 1238–1240. 10.1126/science.1151861 18258860

[CIT0074] Selwyn, B. 2012. “Beyond Firm-Centrism: Re-Integrating Labour and Capitalism into Global Commodity Chain Analysis.” *Journal of Economic Geography* 12 (1): 205–226. 10.1093/jeg/lbr016

[CIT0075] Sills, J., L. Ferrante, and P. M. Fearnside. 2018. “Amazon Sugar Cane: A Threat to the Forest.” *Science* 359 (6383): 1476–1476. 10.1126/science.aat4208 29581381

[CIT0076] Silva, F. N. D. 2013. “Mobilidade urbana: Os desafios do futuro.” *Cadernos Metrópole* 15 (30): 377–388. 10.1590/2236-9996.2013-3001

[CIT0077] Silver, B. J. 2003. *Forces of Labor: Workers’ Movements and Globalization Since 1870*. Cambridge: Cambridge University Press.

[CIT0078] Stattman, S. L., O. Hospes, and A. P. Mol. 2013. “Governing Biofuels in Brazil: A Comparison of Ethanol and Biodiesel Policies.” *Energy Policy* 61: 22–30. 10.1016/j.enpol.2013.06.005

[CIT0079] Stewart, P., and B. Garvey. 2015. “Global Value Chains, Organisations and Industrial Work.” In *Handbook of the Sociology of Work and Employment*, edited by Stephan Edgell, Heidi Gottfried and Edward Granter, 559–575. London, UK: Sage.

[CIT0080] Stewart, P., B. Garvey, M. Torres, and T. Borges De Farias. 2021. “Amazonian Destruction, Bolsonaro and COVID-19: Neoliberalism Unchained.” *Capital & Class* 45 (2): 173–181. 10.1177/0309816820971131 40477074 PMC7689255

[CIT0081] Szmrecsányi, T. 1979. *O planejamento da agroindústria canavieira do Brasil, 1930–1975*. São Paulo: Hucitec.

[CIT0082] Tyfield, D. 2014a. “Putting the Power in ‘Socio-Technical Regimes’–E-Mobility Transition in China as Political Process.” *Mobilities* 9: 585–603. 10.1080/17450101.2014.961262

[CIT0083] Tyfield, D. 2014b. “The Paradoxes of Mass Adoption of EVs: A Socio-Technical Systems Perspective.” IET Hybrid & Electric Vehicle Conference, October 17th 2014. Chongqing: China Automotive Engineering RI.

[CIT0084] Tyfield, D., M. Büscher, M. Freudendal-Pedersen, S. Kesselring, and N. Grauslund Kristensen. 2020. “Chapter 33: Phronesis (and its Potentially Central Contribution to Mobilities Research in the Twenty-First Century).” In *Handbook of Research Methods and Applications for Mobilities*, edited by Monika Büscher, Malene Freudendal-Pedersen, Sven Kesselring and Nikolaj Grauslund Kristensen, 345–353. Cheltenham: Edward Elgar Publishing.

[CIT0085] UNICA. 2019. “Balanço de atividades: 2012/13 a 2018/19.” União da Indústria de Cana de-Açúcar e Bioenergia.

[CIT0086] Valkenburg, G., A. Mamidipudi, P. Pandey, and W. E. Bijker. 2020. “Responsible Innovation as Empowering Ways of Knowing.” *Journal of Responsible Innovation* 7 (1): 6–25. 10.1080/23299460.2019.1647087

[CIT0087] Vargas-Canales, J. M., J. D. J. Brambila-PAZ, V. Pérez-Cerecedo, M. M. Rojas-Rojas, M. D. C. López-Reyna, and J. M. Omaña-Silvestre. 2022. “Trends in Science, Technology, and Innovation in the Agri-Food Sector.” *Tapuya: Latin American Science, Technology and Society* 5 (1): 2115829. 10.1080/25729861.2022.2115829

[CIT0088] VIDAL, M. D. F. 2021. Açúcar: cenário mundial e situação de produção no Brasil e no Nordeste brasileiro.

[CIT0089] Yates, S. 2014. “Power-Knowledge.” In *Encyclopedia of Critical Psychology*, edited by T. Teo, 1480–1485. New York, NY: Springer New York.

